# Extraction and bioactive profile of the compounds produced by *Rhodococcus* sp. VLD-10

**DOI:** 10.1007/s13205-016-0576-6

**Published:** 2016-12-10

**Authors:** Bokka Yellamanda, Muvva Vijayalakshmi, Alapati Kavitha, Dorigondla Kumar Reddy, Yenamandra Venkateswarlu

**Affiliations:** 1 Department of Botany and Microbiology, Acharya Nagarjuna University, Guntur, 522 510 India; 2 Department of Biochemistry, Indian Institute of Science, Bangalore, 560 012 India; 3 Division of Natural Products, Indian Institute of Chemical Technology, Hyderabad, 500 007 India

**Keywords:** Actinobacteria, *Rhodococcus*, Bioactive compounds, 3-Isopropylhexahydro-1H-pyrido[1,2-a] pyrazine-1,4(6H)-dione

## Abstract

A potent actinobacterial strain isolated from the marine samples of Bheemunipatnam beach, Visakhapatnam, India, was identified as *Rhodococcus* sp. VLD-10 using the conventional and genomic (16S rRNA) approaches. Bioactive compounds responsible for the antimicrobial activity of the strain were elucidated by cultivating the strain VLD-10 in a modified yeast extract-malt extract-lactose broth followed by subsequent chromatographic and spectroscopic analyses. Extraction, purification, and structural confirmation of five compounds, viz., benzoic acid, 2-nitrobenzaldehyde, 4-chlorobenzaldehyde, nonadeconoic acid, and 3-isopropylhexahydro-1H-pyrido[1,2-a] pyrazine-1,4(6H)-dione, from *Rhodococcus* sp. VLD-10 were fruitfully described. The bioactivity of the compounds isolated from the strain VLD-10 against Gram-positive as well as Gram-negative bacteria, yeast, and molds was tested and their minimum inhibition concentration was reported. Antibacterial activity of 3-isopropylhexahydro-1H-pyrido[1,2-a] pyrazine-1,4(6H)-dione is more prominent against *Bacillus subtilis*, *B*. *cereus*, *B*. *megaterium*, *Corynebacterium diphtheriae,* and *Escherichia coli,* whereas its antifungal spectrum showed less potency against yeast and fungi. This is the first report on the natural occurrence and bioactivity of 3-isopropylhexahydro-1H-pyrido[1,2-a] pyrazine-1,4(6H)-dione from *Rhodococcus* sp. VLD-10.

**Electronic supplementary material:**

The online version of this article (doi:10.1007/s13205-016-0576-6) contains supplementary material, which is available to authorized users.

## Introduction

Microorganisms are capable of carrying out a tremendous variety of reactions and can adapt to a range of environments allowing them to be transplanted from nature to the laboratory where they can be grown on inexpensive carbon and nitrogen sources to produce valuable compounds (Narayana and Vijayalakshmi 2008; Manivasagan et al. 2013). Because of their biological activity, secondary metabolites of microbial origin are extremely important to our health and nutrition, and have a tremendous economic importance. The screening of microbial natural products continues to represent an important route to the discovery of novel chemicals, for development of new therapeutic agents and for evaluation of the potential of lesser-known or new bacterial taxa (Kurtboke and Wildman 1998; Ramesh and William 2012). Natural products or their derivatives remain the most significant source of novel medicines (Newman et al. 2003; Fenical 2006; Lam 2007; Manivasagan et al. 2013). Among the potential sources of natural products, bacteria are proven to be a predominantly prolific resource with a surprisingly small group of taxa accounting for the vast majority of compounds discovered (Keller and Zengler 2004). Among them, bacteria belonging to the order *Actinomycetales* (commonly called actinobacteria) are distributed ubiquitous in nature and account for more than 50% of the compounds reported in the Dictionary of Natural Products.

In world’s 70% water ecosystem, Indian marine environment is believed to have rich microbial diversity. However, the wealth of indigenous marine microflora has not been fully explored. Most of the studies on marine microorganisms have been limited to isolation, identification, and maintenance of these organisms on different culture media. Their biotechnological potentials are yet to be fully explored (Sivakumar et al. 2007; Manivasagan et al. 2013). East Coast of India is reported to be a major source of actinobacteria (Sambamurthy and Ellaiah 1974; Balagurunathan 1992; Dhanasekaran et al. 2005; Vijayakumar et al. 2007). Therefore, there is tremendous scope to identify new or rare marine microorganisms from this region and also to discover novel microbial metabolites with diverse biological activities (Dhanasekaran et al. 2005; Ramesh and Mathivanan 2009; Ramesh and William 2012). The recent discovery of novel secondary metabolites from taxonomically unique populations of marine actinobacteria suggested that these bacteria add an important new dimension to microbial natural product research. Continued efforts to characterize marine actinobacterial diversity and how adaptations to the marine environment affect secondary metabolite production will create a better understanding of the potential utility of these bacteria as a source of useful products for biotechnology (Jensen et al. 2015). These findings will hopefully encourage additional studies addressing the ecological roles of actinobacteria in the marine environment, their diversity, distribution, culture requirements, and evolutionary responses to life in the sea. In view of the significance of marine actinobacteria as potential producers of bioactive compounds, this study is mainly aimed to identify the potent strain, *Rhodococcus* sp. VLD-10, isolated from marine soil samples of Bheemili beach, Visakhapatnam, India, and to characterize the bioactive metabolites responsible for its antimicrobial activity.

## Materials and methods

### Isolation and identification of an actinobacterial strain VLD-10

An actinobacterial strain, VLD-10, was isolated from marine samples collected at a depth of 5–10 cm from Bheemunipatnam beach, Visakhapatnam, India, in sterile polyethylene bags. The soil dried at 45 °C for 1 h in hot air oven was pretreated with calcium carbonate (1:1 w/w) followed by plating on YMD agar medium using soil dilution technique (Williams and Cross 1971).

### Taxonomic studies of the actinobacterial strain VLD 10

Cultural, physiological, and morphological characteristics together with genomic (16S rDNA gene sequencing) analysis of the strain were studied.

The growth characteristics of the strain were studied on seven International *Streptomyces* Project (ISP) media, such as ISP-1 (Tryptone-yeast extract agar), ISP-2 (Yeast extract-malt extract-dextrose agar), ISP-3 (Oat meal agar), ISP-4 (Inorganic salts starch agar), ISP-5 (Glycerol-asparagine salts agar), ISP-6 (Peptone yeast extract iron agar medium), and ISP-7 (Tyrosine agar), as well as on five nonISP media, such as Nutrient agar (NA), Czapek–Dox (CD) agar, Bennett’s agar, Glucose–tryptone (GT) agar, and Starch casein (SC) agar, with the initial pH 7.2 maintained at 30 °C (Dietz and Theyer 1980). Cultural characters, such as growth, color of the aerial and substrate mycelia, and pigment production, were observed. Physiological and biochemical tests of the strain were examined using standard protocols (Shirling and Gottlieb 1966).

Slide culture technique was employed to study the micro-morphology of the strain cultured on ISP-2 medium. The detailed micro-morphology of the strain was recorded using Scanning Electron Microscopy (SEM) as previously described (Bozzola and Russell 1999). The culture was fixed in 2.5% glutaraldehyde prepared in 0.1 M phosphate buffer (pH 7.2) for 24 h at 4 °C followed by the post-fixation step in 2% aqueous osmium tetroxide for 4 h in the same buffer. The sample was then dehydrated in ethanol and then dried up to critical with the help of Electron Microscopy Science CPD unit (Ruska Labs, Acharya N. G. Ranga Agricultural University, Hyderabad, India). The dried sample was mounted on aluminum stubs covered with double-sided carbon tape. A thin layer of gold coating was applied over the sample using automated sputter coaster for 3 min (JEOL JFC-1600, Japan). Finally, the samples were examined under SEM at various magnifications (Model: JOEL-JSM 5600, Japan).

### Molecular identification of the strain based on 16S rDNA sequence analysis

The strain grown in YMD broth for 3 days was centrifuged at 10,000 rpm for 20 min and the pellet was used for the extraction of DNA (Mehling et al. 1995). PCR mixture consisted of 2.5 μl of 10X Taq buffer, 3.5 μl of MgCl_2_ (25 mM), 2 μl of dNTP (0.4 mM), 1 μl of 16S rDNA forward primer—5′-CCCATG TTGGGTATTCCTCCAGGCGAAAACGGG 3′ (10 pmol/μl), 1 μl of 16S rDNA reverse primer—5′CCCGCATTATCCGTACTCCCCAGGCGGGGC-3′ (10 pmol/μl), Taq polymerase (2 U/μl), and 2 μl template DNA. PCR amplification was carried out as follows: the initial denaturation step at 94 °C for 3 min followed by 30 cycles of denaturation at 94 °C for 1 min, annealing at 65 °C for 1 min, and extension at 72 °C for 1 min, with a further 5 min extension at 72 °C. The PCR product was purified with agarose gel DNA purification kit (SoluteReady^®^ Genomic DNA purification kit, HELINI Biomolecules, Chennai, India) followed by sequencing of 750 bp. The deduced partial 16S rDNA gene sequence was compared with the accessible sequences in GenBank (http://www.ncbi.nlm.nih.gov/) using Basic Local Alignment Search Tool (BLAST) in NCBI GenBank databases. Phylogenetic and molecular evolutionary analyses were conducted using Molecular Evolutionary Genetic Analysis (*MEGA)* version 4.0 (Tamura et al. 2007).

### Extraction, purification, and structural confirmation of bioactive compounds produced by Rhodococcus sp. VLD-10

#### Production of bioactive metabolites

For the large-scale production of bioactive compounds from the strain, 10% of seed broth was inoculated into the optimized production medium (lactose @ 10 g, yeast extract @ 10 g, malt extract @ 10 g, and sodium chloride @ 60 g dissolved in 1 L distilled water and adjusted to pH 7.0) for the enhanced secondary metabolite production. The fermentation was carried out in 1 L Roux bottles for 120 h at 30 °C.

### Isolation, purification, and identification of bioactive compounds

The bioactive compounds from the fermented broth were harvested by filtration of biomass through Whatman filter paper no. 42 (Merck, Mumbai, India). The culture filtrate (25 L) was extracted twice with an equal volume of ethyl acetate and pooled, and the organic layer was concentrated in a Rotovac. The deep brown semi-solid compound (3.8 g) obtained was applied to a silica gel G column (80 × 2.5 cm, Silica gel, Merck, Mumbai, India).

The separation of the crude extract was conducted via gradient elution with hexane: ethyl acetate. The eluent was run over the column and small volumes of eluent collected in test tubes were analyzed via thin-layer chromatography (TLC) using silica gel plates (Silica gel, Merck, Mumbai, India) with hexane: ethyl acetate solvent system. Compounds with identical retention factors (*R*_f_) were combined and assayed for antimicrobial activities. The crude eluent was recuperated in 5–10 ml of ethyl acetate and was further purified.

Among the 11 main fractions eluted, 10 fractions were found polar and 1 was nonpolar residue. Antimicrobial activity was tested for all the fractions obtained. Among the 10 polar fractions eluted from the crude extract, three fractions along with the nonpolar fraction exhibited high antimicrobial activity. D1 (70–30 v/v), D2 (30–70 v/v), D3 (20–80 v/v), and D4 (100–0 v/v) were the fractions collected at different hexane: ethyl acetate solvent system. All the fractions were rechromatographed using different gradient eluent systems for final elucidation of compounds. The fraction D1 on further purification yielded two compounds each in pure form (D1Ba and D1Bb). The second fraction D2 also yielded two subfractions in pure form namely D2Ba and D2Bb. The fraction D3 was single and obtained in pure form. The structure of these active fractions was analyzed on the basis of Fourier Transform Infrared (FTIR); model: Thermo Nicolet Nexus 670 spectrophotometer with NaCl optics, Electron Ionization Mass/Electron Spray Ionization Mass Spectrophotometry (EIMS/ESIMS); model: Micromass VG-7070H, 70 eV spectrophotometer and Nuclear Magnetic Resonance (^1^H NMR and ^13^C NMR); and model: Varian Gemini 200, and samples were made in CDCl_3_ with trimethyl silane as standard.

### Determination of minimum inhibitory concentration (MIC) of bioactive compounds

The antimicrobial spectra of the bioactive compounds produced by the strain were determined in terms of minimum inhibitory concentration (MIC) against a wide variety of Gram-positive, Gram-negative bacteria, and fungi using agar plate diffusion assay (Cappuccino and Sherman 2002). Nutrient agar and Czapek–Dox agar were the media prepared for the growth of bacteria and fungi, respectively. The metabolite dissolved in DMSO at concentrations ranging from 0 to 1000 μg/ml was used to assay against the test bacteria, such as *B. cereus* (MTCC 430), *B. megaterium* (NCIM 2187), *B. subtilis* (MTCC 441), *Corynebacterium diphtheriae* (MTCC 116), *E. coli* (MTCC 40), *Pseudomonas aeruginosa* (MTCC 424), *Salmonella typhi* (ATCC 14028), *Serratia marcescens* (MTCC 118), *Shigella flexneri* (MTCC 1457), *Staphylococcus aureus* (MTCC 96), *Xanthomonas campestris* (NCIM 2310), and fungi, including *Aspergillus niger* (ATCC 1015), *Alternaria alternata* (MTCC 6572), *Botrytis cinerea*, *C. albicans* (MTCC 183), *Fusarium oxysporum* (MTCC 218), *F*. *solani* (MTCC 4634), and *Verticillium alboatrum*. The inoculated plates were examined after 24–48 h of incubation at 37 °C for bacteria and 48–72 h at 28 °C for fungi. The lowest concentration of the bioactive metabolite exhibiting significant antimicrobial activity against the test microbes was taken as the MIC of the compound.

## Results and discussion

### Isolation and identification of an actinobacterial strain VLD-10

An actinobacterial strain, VLD-10, isolated from marine samples of Bheemunipatnam beach, Visakhapatnam, India, was purified on YMD agar medium using classical microbiological methods. Taxonomic position of the strain was described on the basis of cultural, morphological, physiological, and genomic analyses.

**Fig. 1:**
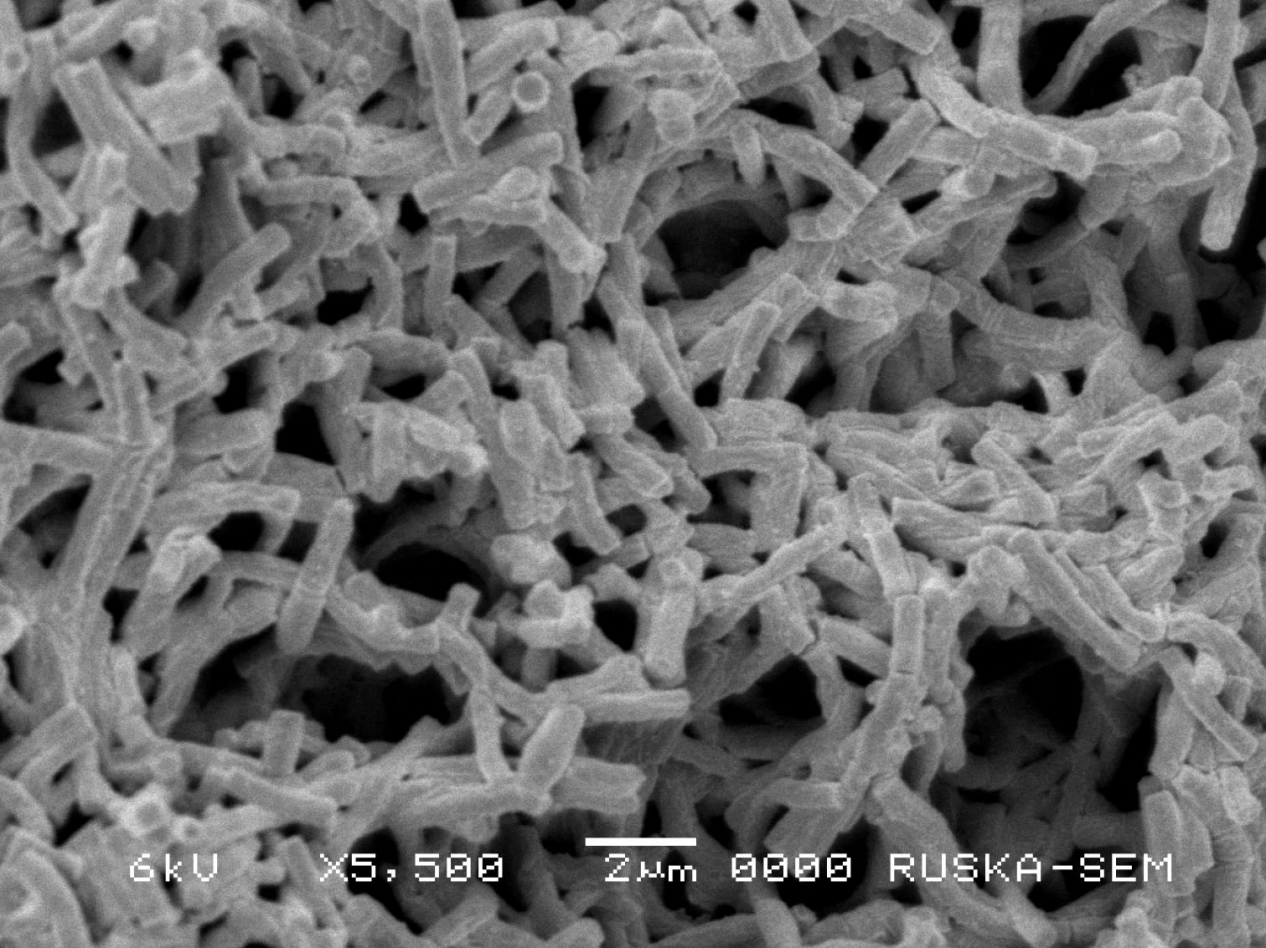
Scanning electron microscopic photograph of the actinobacterial strain VLD 10

Cultural characteristics of the strain were studied by growing the isolate on seven ISP media and five nonISP media and the results are tabulated in Table 1. It exhibited good growth on ISP 2, ISP 4, ISP 6, Bennett’s Agar, and SC Agar, while it was moderate on ISP 1, ISP 3, ISP 5, NAM, CD, and GT agar media. Growth was found to be less on ISP 7. No pigment production was observed in any of the media. Color of aerial and substrate mycelium ranged from dark brown to light brown. Aerial mycelium was white on NAM and CD agar media. Micromorphological studies of the strain through slide culture technique and Scanning Electron Microscopy (SEM) revealed the formation of short rods by the fragmentation of hyphae in their growth phase (Fig. 1).

**Table 1:** Cultural characteristics of the strain VLD10

Culture media	Growth	Color of aerial mycelium	Color of substrate mycelium	Pigment production
ISP-1	Moderate	Brown	Brown	–
ISP-2	Good	Light brown	Light brown	–
ISP-3	Moderate	Light brown	Light brown	–
ISP-4	Good	Light brown	Light brown	–
ISP-5	Moderate	Brown	Dark brown	–
ISP-6	Good	Brown	Dark brown	–
ISP-7	Poor	Light brown	Brown	–
NAM	Moderate	White	Brown	–
CD agar	Moderate	White	Brown	–
Benett’s agar	Good	Brown	Dark brown	–
GT agar	Moderate	Light brown	Brown	–
SC agar	Good	Brown	Dark brown	–

*NAM* nutrient agar medium, *GT agar* glucose–tryptone agar, *CD agar* Czapek–Dox agar, *SC agar* starch casein agar

According to Kampfer et al. (1991), physiological tests play a significant role in the classification and identification of actinobacteria. The strain exhibited good growth with glucose, lactose, starch, and sucrose as carbon sources, while it was moderate with maltose and fructose compared to arabinose, d-galactose, glycerol, mannitol, raffinose, and xylose. It exhibited good growth with organic nitrogen sources like yeast extract and tryptone followed by peptone and l-asparagine. Inorganic nitrogen sources like sodium nitrate, potassium nitrate, and ammonium nitrate did not support the growth. The isolate was indole and methyl red positive. It showed negative results for Voges Proskauer and citrate utilization tests (Table 2). The comparison of biochemical and morphological characteristics of the strain with those reported in Bergey’s Manual of Systematic Bacteriology (Buchannar and Gibbons 1974) revealed its identity as *Rhodococcus* sp.

**Table 2:** Physiological and biochemical characteristics of the strain VLD 10

	Inference
Utilization of carbon sources (w/v)	
Glucose	+++
Lactose	+++
Starch	+++
Sucrose	+++
Fructose	++
Maltose	++
Arabinose	+
Galactose	+
Glycerol	+
Mannitol	+
Raffinose	+
Xylose	+
Utilization of nitrogen sources (w/v)	
Tryptone	+++
Yeast extract	+++
l-Asparagine	++
Peptone	++
Ammonium nitrate	−
Potassium nitrate	−
Sodium nitrate	−
Tyrosine	−
Biochemical characteristics	
Methyl red	P
Indole	P
Voges Proskaeur	N
Citrate utilization	N
H_2_S production	P
Sodium chloride tolerance (w/v)	
0%	−
1–3%	+
4–6%	++
7–8%	+++
Above 8%	+
Enzymatic activity	
Amylase	P
Cellulase	P
Chitinase	P
Pectinase	P
l-Asparaginase	P
Protease	P
RNAase	N
Nitrate reductase	N
DNAase	N
Keratinase	N
Antibiotic sensitivity (µg/ml))	
Ampicillin (10)	S
Chloramphenicol (10)	S
Gentamicin (30)	R
Neomycin (15)	S
Penicillin (25)	S
Rifampicin (20)	S
Streptomycin (50)	R
Tetracycline (35)	S
Vancomycin (30)	R

*P* positive, *N* negative, *S* sensitive, *R* resistant

+++ good growth, ++ moderate growth, + weak growth, − no growth

The strain exhibited good growth in the medium amended with 7–8% (w/v) NaCl, while growth was moderate between 1 and 6% (w/v). No growth was found in the medium without NaCl indicating its halophilic nature. It could produce a broad range of commercially important enzymes like amylase, cellulase, chitinase, l-asparaginase, protease, and pectinase, but it was negative for DNase, RNase, keratinase, and nitrate reductase. It was found to be sensitive to antibiotics, such as ampicillin, chloramphenicol, neomycin, penicillin, rifampicin, and tetracycline, but showed resistance to gentamicin, streptomycin, and vancomycin (Table 2).

**Fig. 2:**
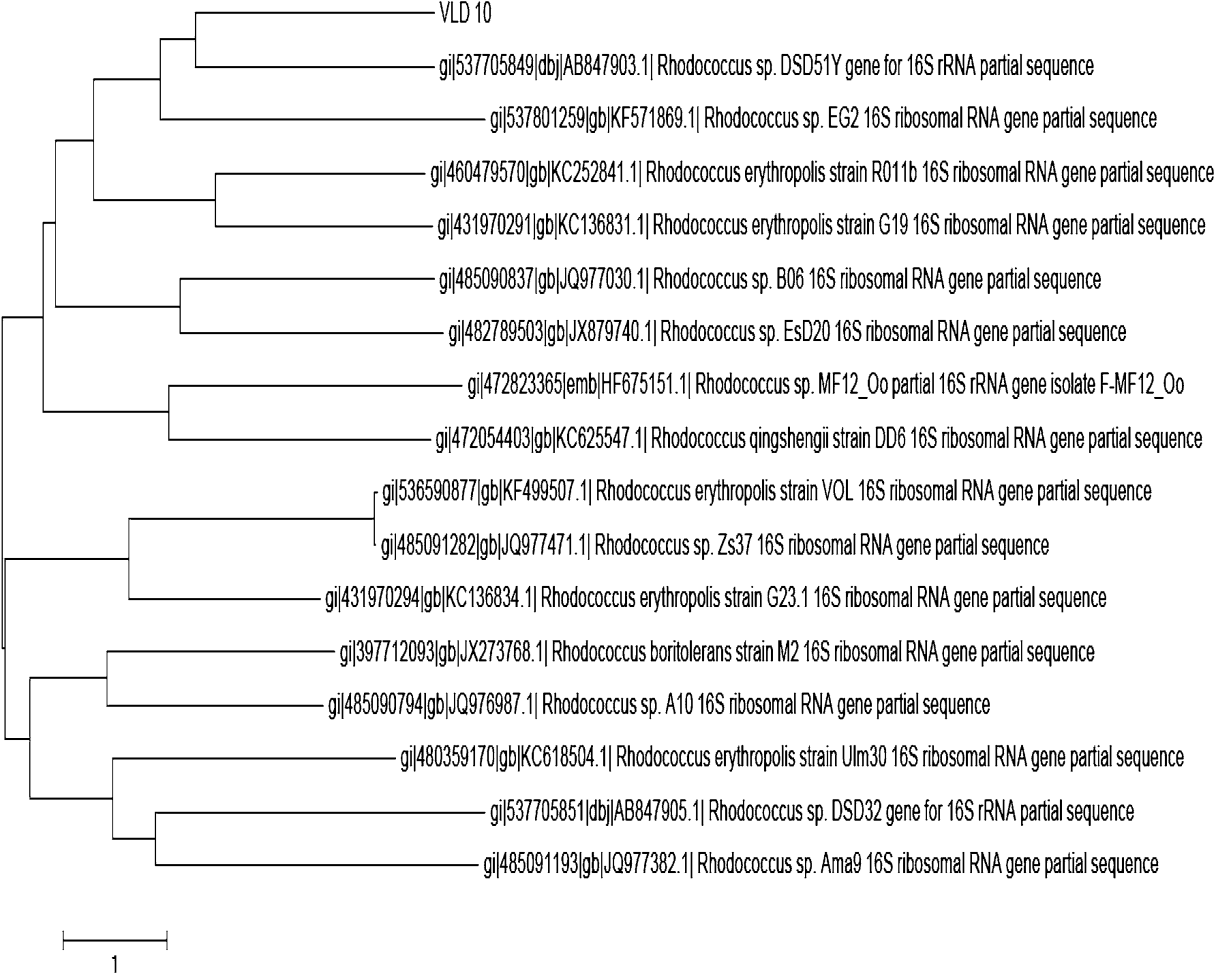
Phylogenetic tree of 16SrRNA sequence of the actinobacterial strain VLD 10 constructed in comparison with those of species of genus *Rhodococcus* using neighbour-joining method. *Bar*, one substitutions per nucleotide position

Using 16S rRNA analysis, the gene sequence of the strain was compared and aligned with those sequences retrieved from NCBI GenBank database using the BLAST analysis. The phylogenetic tree was constructed by neighbour-joining method using the MEGA software (Fig. 2) and deposited in the Gene Bank with an accession number KC505180. Based on these cultural, morphological, physiological, and molecular analyses, the strain VLD-10 was identified as *Rhodococcus* sp. VLD-10 belonging to the family Corynebacteriaceae.

### Isolation and purification of bioactive compounds from crude extract

For the isolation and purification of bioactive compounds, crude extract was applied on a silica gel G column (80 × 2.5 cm, Silica gel, Merck, Mumbai, India) and their separation was conducted via gradient elution with hexane: ethyl acetate. Elutions were collected sequentially in small test tubes and those fractions having similar retention factors (*R*_f_) on thin-layer chromatography (TLC) silica gel plates were pooled together. Out of 11 eluted main fractions, 10 are polar residues and 1 is nonpolar residue. Among the ten polar fractions, three fractions and the single nonpolar fraction exhibited high antimicrobial activity. These four fractions, namely D1, D2, D3, and D4, were collected at different eluent systems of hexane: ethyl acetate solvent systems, viz., 70–30 v/v, 30–70 v/v, 20–80 v/v, and 100–0 v/v, respectively. All the fractions were rechromatographed using different gradient eluent systems to obtain the fractions in pure form for structural elucidation. The flowchart showing the isolation and purification of bioactive fractions is illustrated in Fig S1.

### Physico-chemical properties and structural elucidation of six bioactive compounds

D1 fraction was rechromatographed into two pure fractions, (D1Ba) and (D1Bb). The fraction D1Ba appeared as yellow solid which was soluble in CHCl_3_, MeOH, DCM, and DMSO. The IR absorption maxima *V*_max_ at 1686 cm^−1^ suggested the presence of functional groups like carbonyl groups (Fig. S2a). In ESIMS, the compound showed molecular ions at *m/z* = 123 [M+] inferring the molecular weight of C_7_H_6_O_2_ [M]^+^ (Fig. S2b). The proton NMR of the compound displayed proton signals at signals at δ 8.12–8.15 (m, 2H), 7.59–7.62 (m, 1H), and 7.44–7.46 (m, 2H) due to five aromatic protons (Fig. S2c). ^13^C NMR depicted peak and showed signal at δ 172.7 for carbonyl group (Fig. S2d). Based on these spectral data, the first active fraction (D1Ba) was identified as benzoic acid.

D1Bb fraction soluble in MeOH, DCM, and DMSO appeared as white crystalline powder. The IR absorption maxima *V*_max_ at 1705 cm^−1^ suggested the presence of functional aldehyde (Fig. S3a). In ESIMS, the compound showed molecular ions at *m/z* = 301[2M-1] inferring the molecular weight of 2(C_7_H_5_O_3_N)-1 [2M-1] (Fig. S3b). The proton NMR of the compound displayed proton signals at δ 10.43 (1H, s), and one proton for aldehyde, at δ 8.13 (d, 1H, *J* = 7.7, 1.3 Hz), 7.96 (dd, 1H, *J* = 7.3, 1.9 Hz), and 7.75–7.83 (2H, m) for four aromatic protons (Fig. S3c). ^13^C NMR depicted peaks at δ (188.1) for aldehyde carbon (Fig. S3d). Based on the spectral data, the fraction D1Bb was identified as 2-nitrobenzaldehyde.

D2 fraction was rechromatographed into two pure fractions, (D2Ba) and (D2Bb). D2Ba fraction appeared as white crystalline powder and was soluble in CHCl_3_, MeOH, and DCM. The IR absorption maxima *V*_max_ at 1683 cm^−1^ suggested the presence of aldehyde (Fig. S4a). In ESIMS, the compound showed molecular ions at *m/z* = 141 [M+1]^+^ inferring the molecular weight of C_7_H_6_OCl [M+1]^+^ (Fig. S4b). The proton NMR of the compound displayed proton signals at δ 9.99 (s, 1H) for one aldehyde proton, and at δ 7.83 (d, 2H, *J* = 8.5 Hz) and 7.53 (d, 2H, *J* = 8.5 Hz) for four aromatic protons (Fig. S4c). ^13^C NMR depicted peak at δ 190.7 for aldehyde (Fig. S4d). D2Ba was identified as 4-chlorobenzaldehyde based on the spectral data.

The second fraction D2Bb in pure form appeared as light green liquid soluble in CHCl_3_, MeOH, DCM, and DMSO. The IR absorption maxima *V*_max_ at 1709 cm^−1^ suggested the presence of carboxylic group (Fig. S5a). In ESIMS, the compound showed molecular ions at *m/z* = 299 [M+1]^+^ inferring the molecular weight of C_19_H_38_O_2_ [M+1]^+^ (Fig. S5b). The proton NMR of the compound displayed at δ 2.35 (t, 2H, *J* = 7.2 Hz) for alpha methylene protons; at δ 1.65–1.55 (30H, m) and 1.25–1.99 (m, 2H) for aliphatic methylene protons; at δ 1.25–1.99 (m, 2H) for methylene protons; and at δ 0.82 (t, 3H, *J* = 6.1 Hz) for methyl protons (Fig. S5c). ^13^C NMR depicted peak at δ 180.8 for carboxylic group (Fig. S5d). Based on spectral data, the D2Bb fraction was identified as nonadeconoic acid.

Fraction D3 appeared as white solid, which was soluble in CHCl_3_, MeOH, DCM, and DMSO. The IR absorption maxima *V*_max_ at 1687 cm^−1^ suggested the presence of functional groups like carbonyl group (Fig. S6a). In ESIMS, the compound showed molecular ions at *m/z* = 211.1474 [M+1] inferring the molecular weight of C_11_H_19_0_2_N_2_[M+1]^+^ (Fig. S6b). The proton NMR of the compound displayed proton signals at δ 5.91 (s, 1H) for amide protons; 4.12 (t, 1H, *J* = 7.5 Hz) for methylene protons; 4.02 (d, 1H, *J* = 6.7 Hz) for methylene protons; 3.67–3.48 (m, 2H) for methylene protons; 2.45–2.24 (m, 1H) and 2.23–1.97 (m, 3H) for methylene proton; 1.96–1.83 (m, 1H), 1.82–1.69 (m,1H), and 1.60–1.45 (m, 1H) for methylene protons; and 1.05 (d, 3H, *J* = 6.0 Hz) and 0.96 (d, 3H, *J* = 6.0 Hz) for methyl protons. (Fig. S6c). ^13^C NMR depicted peaks at δ 170.2 (1C), 166.1 (1C), 76.5 (1C), 58.9 (1C), 53.3 (1C), 45.4 (1C), 38.5 (1C), 29.6 (1C), 28.0 (1C), 24.6 (1C), 23.2 (1C), 22.7 (1C), and at δ 21.1 (1C) (Fig. S6d). DEPT spectrum exhibiting methyl groups (12, 12^1^), methylene groups (3, 4, 5, 10), and methylene groups (6, 9, 11) (Fig. S6e). ^1^H–^1^H COSY NMR spectrum exhibits correlation between H_12_–H_11,_ H_10_–H_11,_ H_10_–H_9,_ H_6_–H_5,_ H_5_–H_4,_ H_4_–H_3_ (Fig. S6f). HSQC spectrum exhibits correlation between 13 C NMR with ^1^H NMR: C_3_–H_3_, C_4_–H_4_, C_5_–H_5_, C_6_–H_6_, C_9_–H_9_, C_10_–H_10_, C_11_–H_11_, C_12_–H_12_, and C_13_–H_13_ (Fig. S6g). HMBC spectrum exhibits following correlation of 13 C NMR with ^1^H NMR 23.2–1.05; 21.1–0.96; 24.6–1.55; 38.5–1.55, 2.08; 53.3–4.03; 58.9–4.15; 28.0–2.18, 2.38; 22.7–1.97, 2.05; 45.4–3.60; 170–4.12; and 166.1–4.02. (Fig. S6h). Based on these spectral data, the active fraction D3 was identified as 3-isobutylhexahydropyrrolo[1,2-a]pyrazine-1,4-dione This is the first report from actinobacteria.

### Determination of MIC of the isolated bioactive compounds

**Fig. 3:**
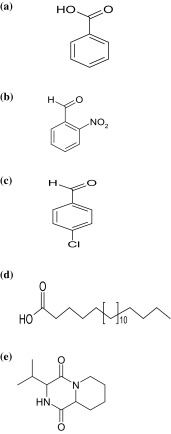
**a** Molecular structure of benzoic acid, **b** molecular structure of 2-nitrobenzaldehyde, **c** molecular structure of 4-chlorobenzaldehyde, **d** Molecular structure of nonadeconoic acid, and **e** molecular structure of 3-isopropylhexahydro-1H-pyrido[1,2-a] pyrazine-1,4(6H)-dione

MIC of the bioactive compounds, viz., benzoic acid, 2-nitrobenzaldehyde, 4-chlorobenzaldehyde, nonadeconoic acid, and 3-isopropylhexahydro-1H-pyrido[1,2-a] pyrazine-1,4(6H)-dione (Fig. 3), along with crude extract of the strain VLD-10 against bacteria and fungi was determined. The sensitivity of bacteria as well as fungi to the compounds exhibited variation and the MIC of these compounds ranged from 5–100 µg/ml (Table 3). Bioactive compounds of the crude extract showed good antimicrobial activity against test bacteria and fungi in the range of 10–20 µg/ml. Benzoic acid (D1Ba) is active against bacteria, such as *B. cereus, S. aureus,* and *X. campestris* as compared to the other bacteria tested, while *Botrytis cinerea* is sensitive among fungi. 2-nitrobenzal dehyde (D1Bb) is mostly active against bacteria like *B. subtilis, Shigella flexneri,* and *Staphylococcus aureus*. 4-chlorobenzaldehyde (D2Ba) is active against *S. aureus, B. subtilis,* and *S. flexneri*. Nonadeconoic acid (D2Bb) is active against *S. flexneri*, *S. aureus, B. subtilis,* and *X. campestris*. Among all the compounds, 3-isopropylhexahydro-1H-pyrido[1,2-a] pyrazine-1,4(6H)-dione (D3) is more active against *B*. *subtilis*, *B*. *cereus*, *B*. *megaterium*, *C*. *diphtheriae,* and *E*. *coli,* but it showed less activity against the fungi when compared to that of standard control (nystatin). The partially purified fraction (D4) exhibited good activity against *Bacillus* spp. tested.

**Table 3:** Minimum inhibitory concentration (MIC) of the bioactive compounds produced by *Rhodococcus* sp. VLD10

Test organism	MIC (µg/ml)						
	D1Ba	D1Bb	D2Ba	D2Bb	D3	Crude extract	Positive control
Bacteria							
* Bacillus cereus*	20	40	35	35	20	10	15
* Bacillus megaterium*	45	35	45	55	20	15	25
* Bacillus subtilis*	35	20	20	20	15	10	20
* Corynebacterium diphtheriae*	30	45	55	55	20	10	30
* Escherichia coli*	30	35	45	35	25	10	20
* Pseudomonas aeruginosa*	40	45	35	40	45	10	40
* Salmonella typhi*	50	50	45	45	50	20	45
* Serratia marcescens*	30	35	30	35	25	10	25
* Shigella flexneri*	35	20	20	15	25	15	15
* Staphylococcus aureus*	25	25	15	15	30	10	20
* Xanthomonas campestris*	25	35	25	20	25	10	25
Fungi							
* Aspergillus niger*	50	85	95	70	50	20	5
* Botrytis cinerea*	25	80	90	75	45	15	10
* Candida albicans*	55	95	100	90	40	15	10
* Fusarium oxysporum*	50	80	70	65	35	20	10
* Fusarium solani*	35	100	80	65	40	20	10
* Verticillium alboatrum*	80	90	80	70	55	20	10
* Alternaria alternata*	65	75	75	85	40	20	5

Tetracylcline is the positive control for bacteria and Nystatin for fungi

*5D1Ba* benzoic acid, *D2Ba* 4-chlorobenzaldehyde, *D1Bb* 2-nitrobenzaldehyde, *D2Bb* nonadeconoic acid, *D3* 3-isopropylhexahydro-1H-pyrido[1,2-a] pyrazine-1,4(6H)-dione

Rhodococci are notable for the ability to degrade a variety of natural and xenobiotic compounds (Bell et al. 1998) along with few bioactive metabolite reports. Chiba et al. (1999) purified and elucidated a novel antifungal cyclic tetrapeptide, Rhodopeptins (Mer-N1033) from *Rhodococcus* sp. Two antimycobacterial agents, lariatins A and B, were elucidated by Iwatsuki et al. (2006) from the culture broth of *Rhodococcus* sp. K01-B0171 using spectral analysis and advanced protein chemical methods. Kitagawa and Tamura (2008) isolated a new quinoline antibiotic, aurachin RE from the culture broth of *Rhodococcus erythropolis* JCM 6824 active against both high- and low-GC Gram-positive bacteria.

Isolation and purification of rhodostreptomycin A and B by a combination of cation exchange (CM-Sephadex) and reversed-phase HPLC (Lichrospher 60RP-select B) from the culture broths of *Rhodococcus fascians* 307CO were recorded (Kurosawa et al. 2008). Abdel-Meged et al. (2011) purified, characterized, and tested the antimicrobial activity of glycolipids produced by *Rhodococcus erythropolis* isolated from soils of Riyadh area, Saudi Arabia. Borisova (2011) found that antimicrobial compound (MW 911.5452 Da) of *Rhodococcus opacus* isolated from the soils of East Tennessee State University inhibited *R. erythropolis* and a large number of closely related species.

This study has revealed the production of five bioactive compounds, such as benzoic acid, 2-nitrobenzaldehyde, 4-chlorobenzaldehyde, nonadeconoic acid, and 3-isopropylhexahydro-1H-pyrido[1,2-a] pyrazine-1,4(6H)-dione, from *Rhodococcus* sp. VLD-10. Benzoic acid is a well-known food preservative that inhibits the growth of bacteria, yeasts, and molds (Warth 1991), which is also evident from our findings in vitro. The bioactive compounds, 2-nitrobenzaldehyde and 4-chlorobenzaldehyde play a prominent role in the manufacture of pharmaceuticals, dyes, agrochemicals, and other organic compounds. However, their natural occurrence from *Rhodococcus* spp. and their biological significance is not yet reported. This is the first report of 3-isopropylhexahydro-1H-pyrido[1,2-a] pyrazine-1,4(6H)-dione isolated from *Rhodococcus* sp. VLD-10.

## Electronic supplementary material

Below is the link to the electronic supplementary material.

Supplementary material 1 (DOC 1321 kb)

## References

[CR1] Abdel-Meged A, Al-Rahama AN, Mostafa AA, Husnu Can Baser K (2011). Biochemical characterization of antimicrobial activity of glycolipids produced by *Rhodococcus erythropolis*. Pak J Bot.

[CR2] Balagurunathan R (1992) Antagonistic actinomycetes from Indian shallow sea sediments with reference to α, β-unsaturated γ-lactone type of antibiotic from *Streptomyces griseobrunneus*. Dissertation, Annamalai University, India

[CR3] Bell KS, Philp JC, Aw DWJ, Christofi N (1998). The genus *Rhodococcus*. J Appl Microbiol.

[CR4] Borisova RB (2011) Isolation of a *Rhodococcus* soil bacterium that produces a strong antibacterial compound. Electronic theses and dissertations, Tennessee State University. http://dc.etsu.edu/etd/1388

[CR5] Bozzola JJ, Russell LD (1999). Electron Microscopy Principles and Techniques for Biologists.

[CR6] Buchannar E, Gibbons NE (1974). Bergey’s manual of determinative bacteriology.

[CR40] Cappuccino JG, Sherman N (2002) Microbiology: a laboratory manual. Benjamin, Harlow, pp 263–264

[CR7] Chiba H, Agematu H, Kaneto R, Terasawa T, Sakai K, Dobashi K, Yoshioka T (1999). Rhodopeptins (Mer-N1033), novel cyclic tetrapeptides with antifungal activity from *Rhodococcus* sp. I. Taxonomy, fermentation, isolation, physico-chemical properties and biological activities. J Antibiot.

[CR8] Dhanasekaran D, Panneerselvam A, Thajuddin N (2005). Antibacterial actinomycetes in marine soils of Tamil Nadu. Geobios.

[CR9] Dietz A, Theyer DW (1980). Actinomycete taxonomy.

[CR10] Fenical W (2006). Marine pharmaceuticals past, present and future. Oceanography.

[CR11] Iwatsuki M, Tomoda H, Uchida R, Gouda H, Hirono S, Oh mura Lariatins S (2006). Antimycobacterial peptides produced by *Rhodococcus* sp. K01-B0171 have a lasso structure. J Am Chem Soc.

[CR12] Jensen PR, Moore BS, Fenical W (2015). The marine actinomycete genus *Salinispora*: a model organism for secondary metabolite discovery. Nat Prod Rep.

[CR13] Kampfer P, Kroppenstedt RM, Dott W (1991). A numerical classification of the genera *Streptomyces* and *Streptoverticillium* using miniaturized physiological tests. J Gen Microbiol.

[CR14] Keller M, Zengler K (2004). Tapping into microbial diversity. Nat Rev.

[CR15] Kitagawa W, Tamura T (2008). Three types of antibiotics produced by *Rhodococcus erythropolis* strains. Microbes Environ.

[CR16] Kurosawa K, Ghiviriga I, Sambandan TG, Lessard PA, Barbara JE, Rha C, Sinskey AJ (2008). Rhodostreptomycins, antibiotics biosynthesized following horizontal gene transfer from *Streptomyces padanus* to *Rhodococcus fascians*. J Am Chem Soc.

[CR17] Kurtboke DJ, Wildman HG (1998). Accessing Australian biodiversity towards an improved detection of actinomycetes—an activity report. Actinomycetes.

[CR18] Lam KS (2007). New aspects of natural products in drug discovery. Trends Microbiol.

[CR19] Manivasagan P, Venkatesan J, Sivakumar K, Klim SK (2013). Marine actinobacterial metabolites: current status and future perspectives. Microbiol Res.

[CR20] Mehling A, Wehmeier UF, Pipersberg W (1995). Nucleotide sequence of the streptomycete 16S ribosomal DNA: towards a specific identification system for streptomycetes using PCR. Microbiol.

[CR21] Narayana KJP, Vijayalakshmi M (2008). Optimization of antimicrobial metabolites production by *Streptomyces albidoflavus*. Res J Pharmacol.

[CR22] Newman DJ, Cragg GM, Snader KM (2003). Natural products as sources of new drugs over the period 1981–2002. J Nat Prod.

[CR23] Ramesh S, Mathivanan N (2009). Screening of marine actinomycetes isolated from the Bay of Bengal, India for antimicrobial activity and industrial enzymes. World J Microbiol Biotech.

[CR24] Ramesh S, William A (2012). Marine actinomycetes: an ongoing source of novel bioactive metabolites. Microbiol Res.

[CR25] Sambamurthy K, Ellaiah P (1974). A new streptomycete producing neomycin (B and C) complex—*S*. *marinensis* Part I. Hindustan Antibiot Bull.

[CR26] Shirling EB, Gottlieb D (1966). Methods for characterization of *Streptomyces* species. Int J Syst Bacteriol.

[CR27] Sivakumar K, Sahu MK, Thangaradjou T, Kannan L (2007). Research on marine actinobacteria in India. Ind J Microbiol.

[CR28] Tamura K, Dudley J, Nei M, Kumar S (2007). MEGA4: molecular evolutionary genetics analysis (MEGA) software version 4.0. Mol Biol Evol.

[CR29] Vijayakumar R, Muthukumar C, Thajuddin N, Panneevselvan A, Saravanamuthu R (2007). Studies on the diversity of actinomycetes in the Palk Strait region of Bay of Bengal, India. Actinomycetologica.

[CR31] Warth AD (1991). Mechanism of action of benzoic acid on *Zygosaccharomyces bailii*: effects on glycolytic metabolite levels, energy production and intracellular pH. Appl Environ Microbiol.

[CR32] Williams ST, Cross T (1971). Isolation, purification, cultivation and preservation of actinomycetes. Methods Microbiol.

