# Supplementary material for: Extraction and bioactive profile of the compounds produced by Rhodococcus sp. VLD-10
Source: 3 Biotech. 2016 Dec 10;6(2):261. doi: 10.1007/s13205-016-0576-6 (PMC5149122; doi:10.1007/s13205-016-0576-6)
Supplement: Supplementary file 1 — Supplementary material 1 (DOC 1321 kb) [file 13205_2016_576_MOESM1_ESM.doc]

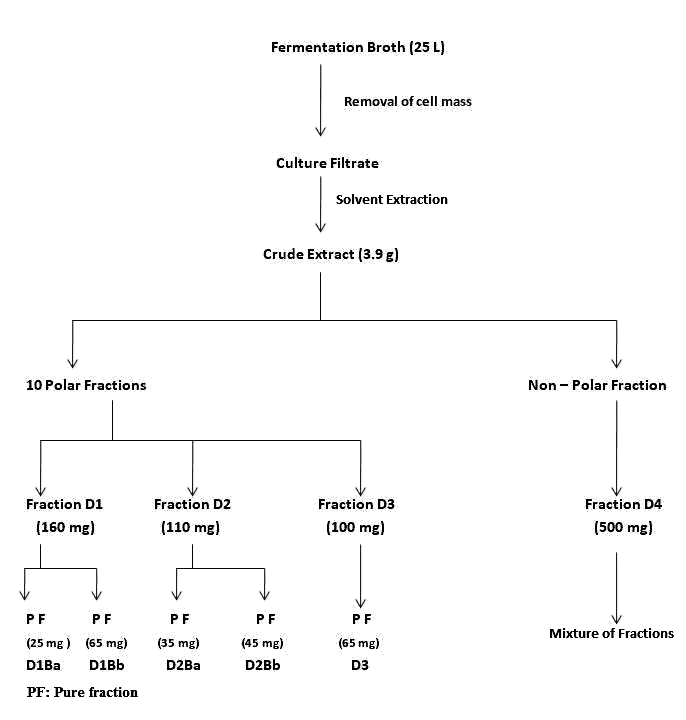


**Fig. S1** Flow chart illustrating isolation and purification of bioactive compounds produced by *Rhodococcus* sp. VLD 10

* D1, D2 and D3 fractions showing antimicrobial activity were used for structural elucidation.

71


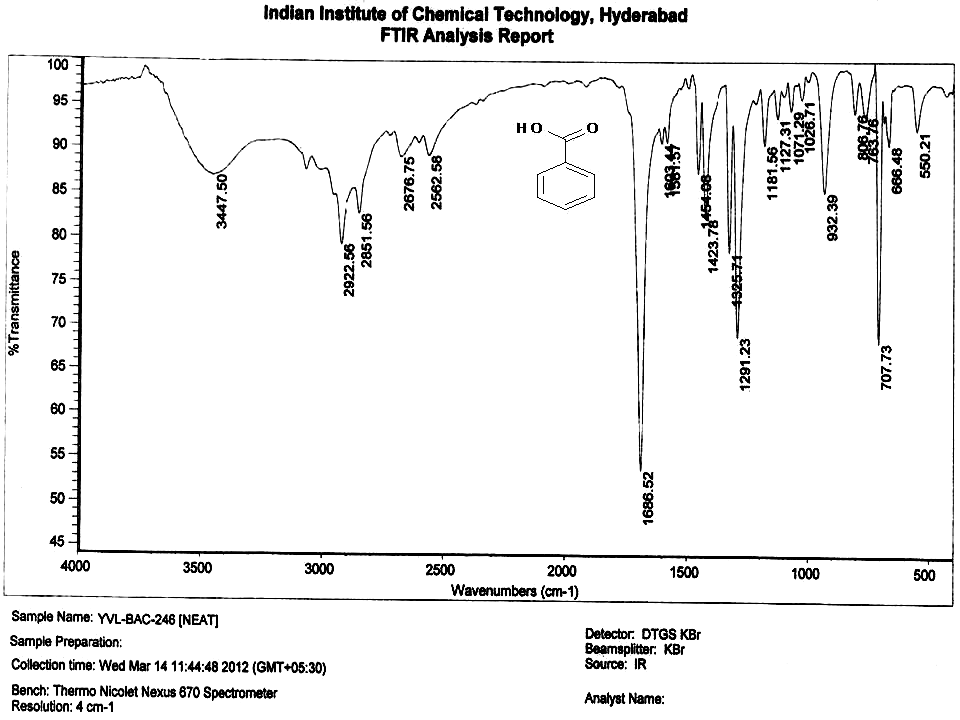


**Fig. S2a** FTIR spectrum of the compound D1Ba produced by *Rhodococcus* sp. VLD 10


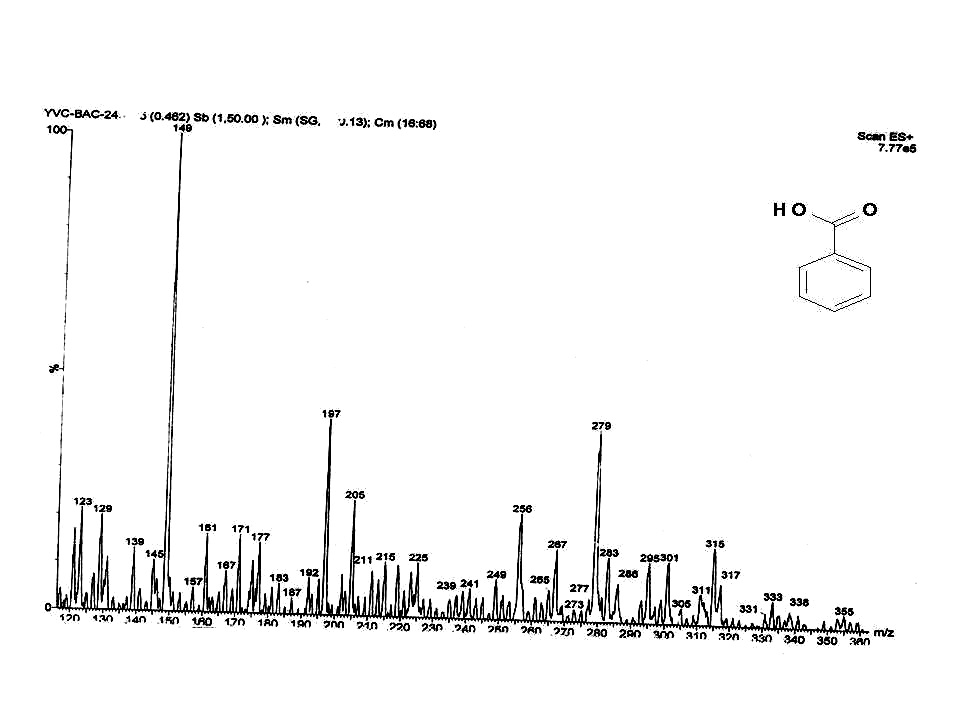


**Fig. S2b** Mass spectrum of the compound D1Ba produced by *Rhodococcus* sp. VLD 10

**
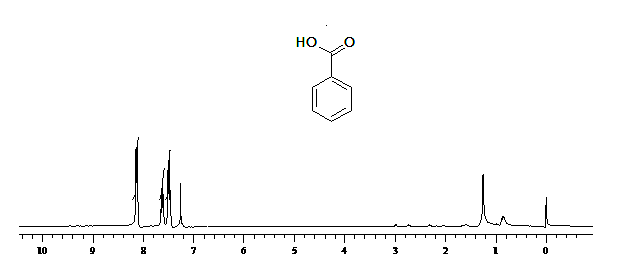
**

**Fig. S2c** ^1^H NMR spectrum of compound D1Ba produced by *Rhodococcus* sp. VLD 10


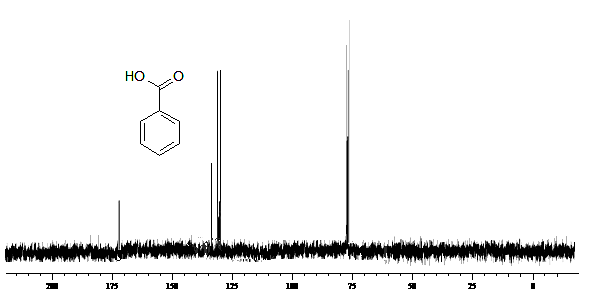


**Fig. S2d** ^13^C NMR spectrum of compound D1Ba produced by *Rhodococcus* sp. VLD 10


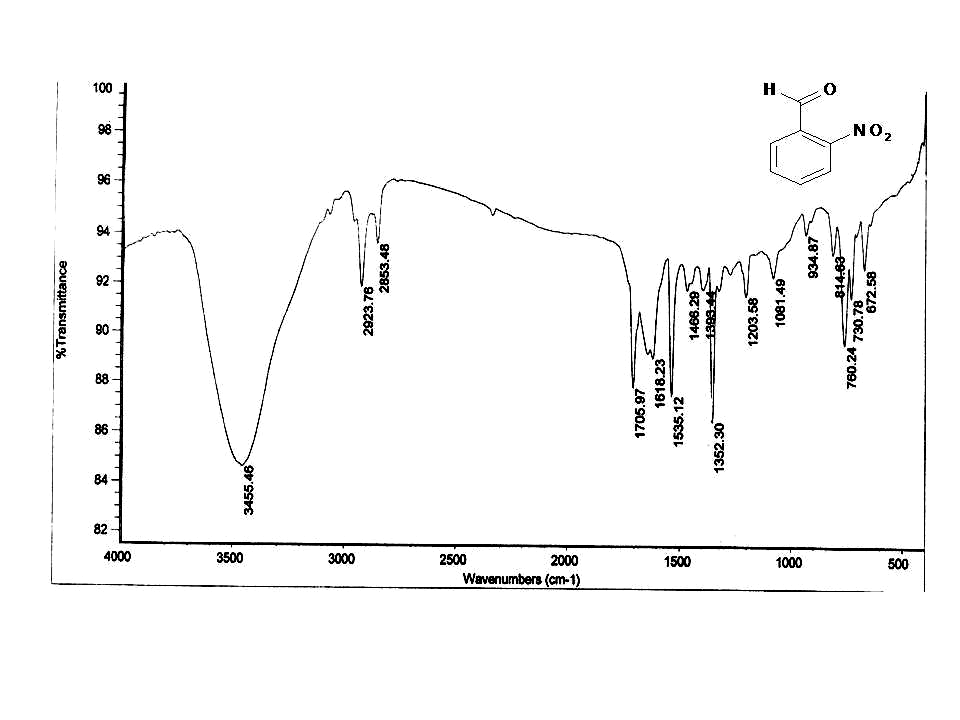


**Fig. S3a** FTIR spectrum of the compound D1Bb produced by *Rhodococcus* sp. VLD 10


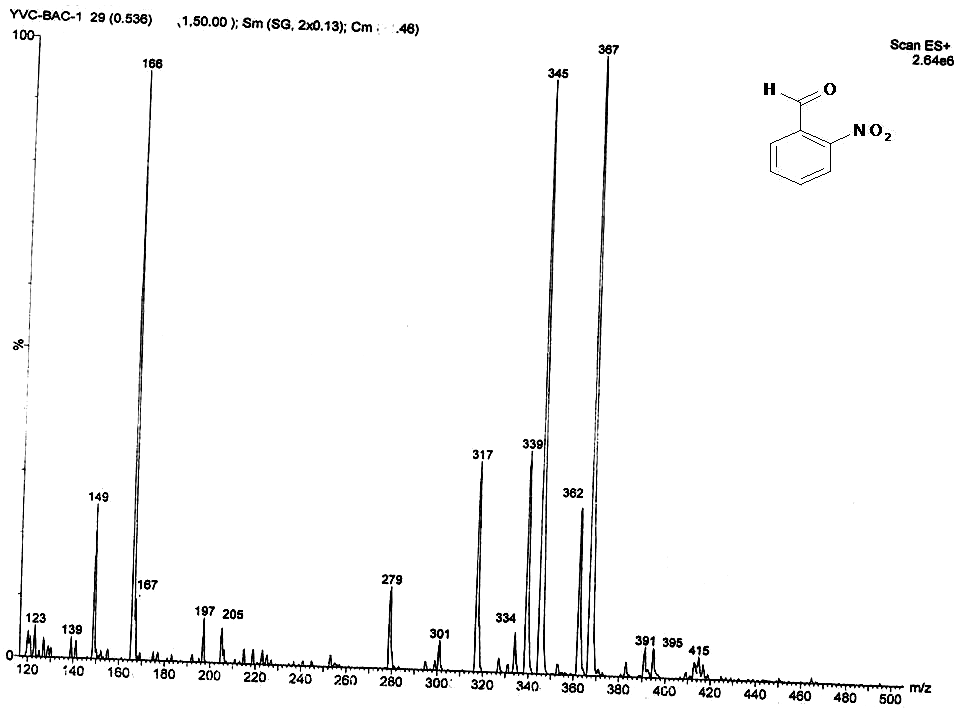


**Fig. S3b** Mass spectrum of the compound D1Bb produced by *Rhodococcus* sp. VLD 10


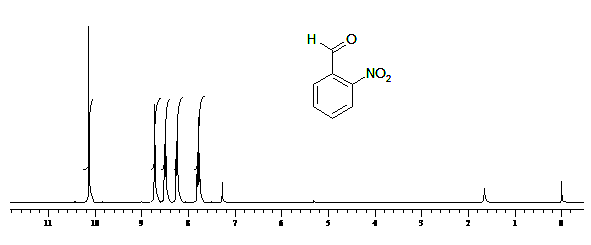


**Fig. S3c** ^1^H NMR spectrum of compound D1Bb produced by *Rhodococcus* sp. VLD 10


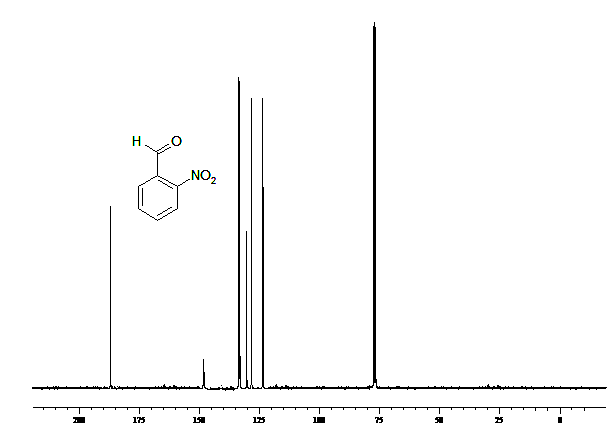


**Fig. S3d** ^13^C NMR spectrum of compound D1Bb produced by *Rhodococcus* sp. VLD 10


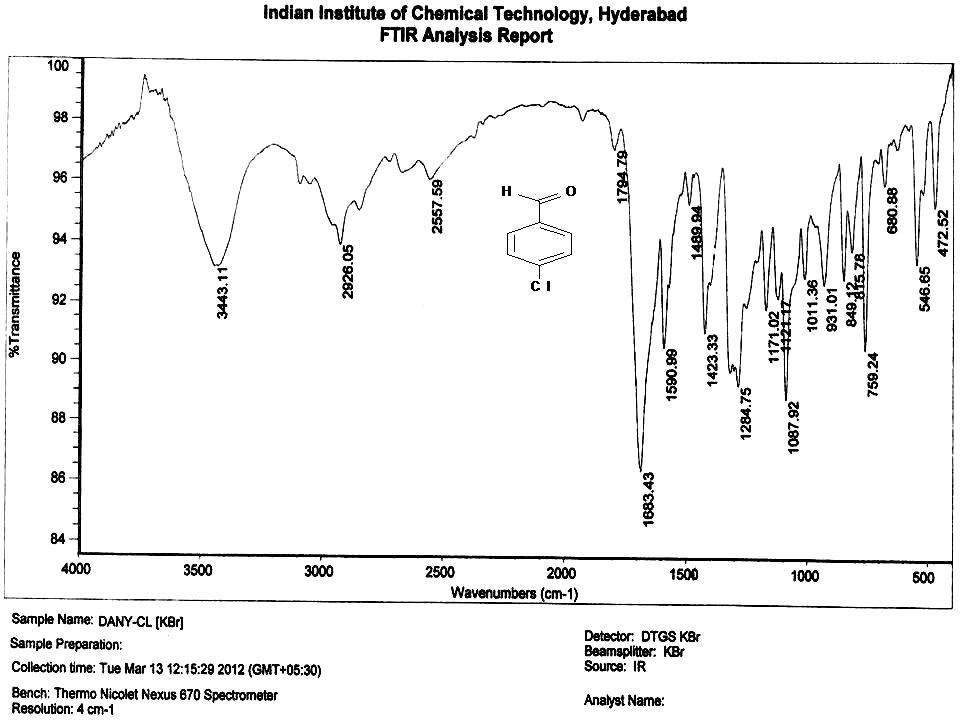


**Fig. S4a** FTIR spectrum of the compound D2Ba produced by *Rhodococcus sp.* VLD 10


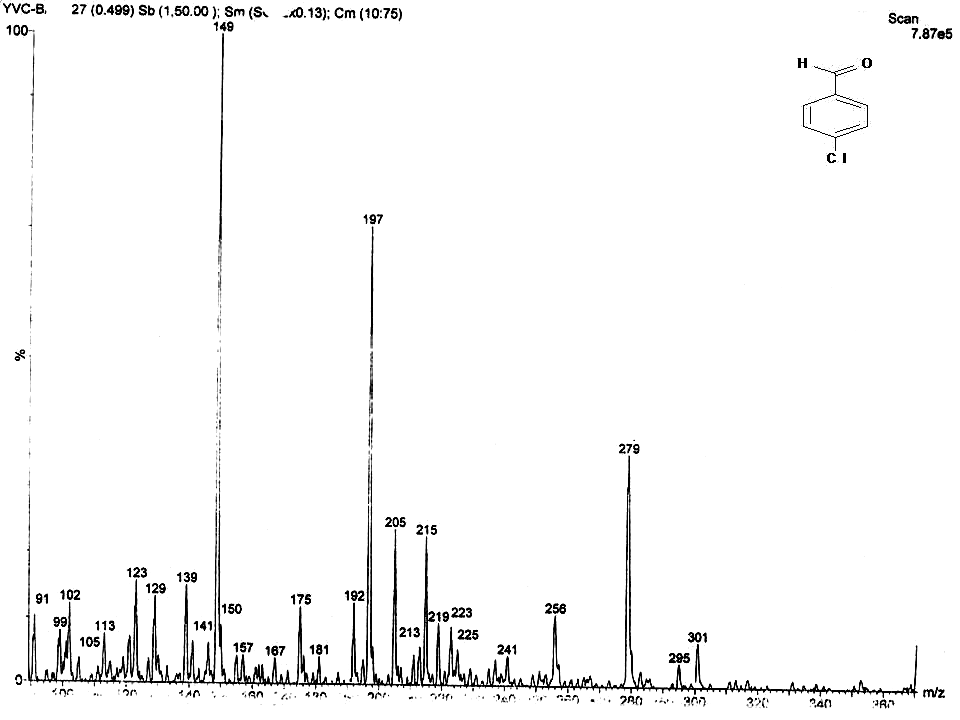


**Fig. S4b** Mass spectrum of the compound D2Ba produced by *Rhodococcus* sp. VLD 10


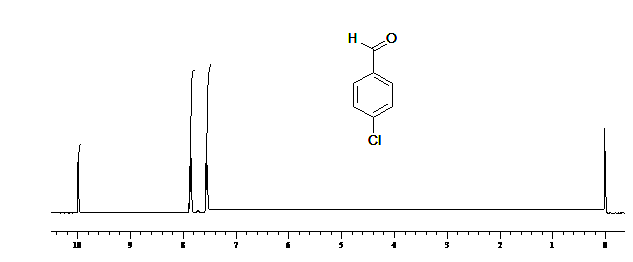


**Fig. S4c** ^1^H NMR spectrum of compound D2Ba produced by *Rhodococcus* sp. VLD 10


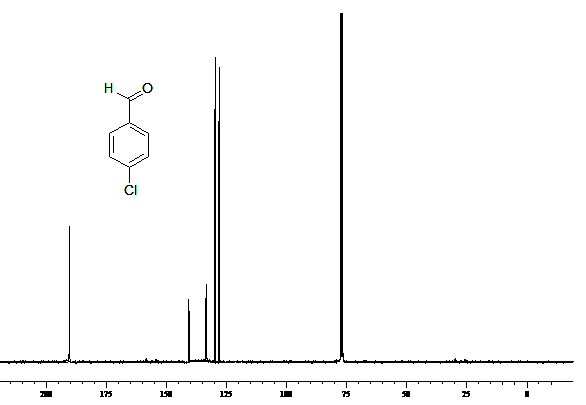


**Fig. S4d** ^13^C NMR spectrum of compound D2Ba produced by *Rhodococcus* sp. VLD 10


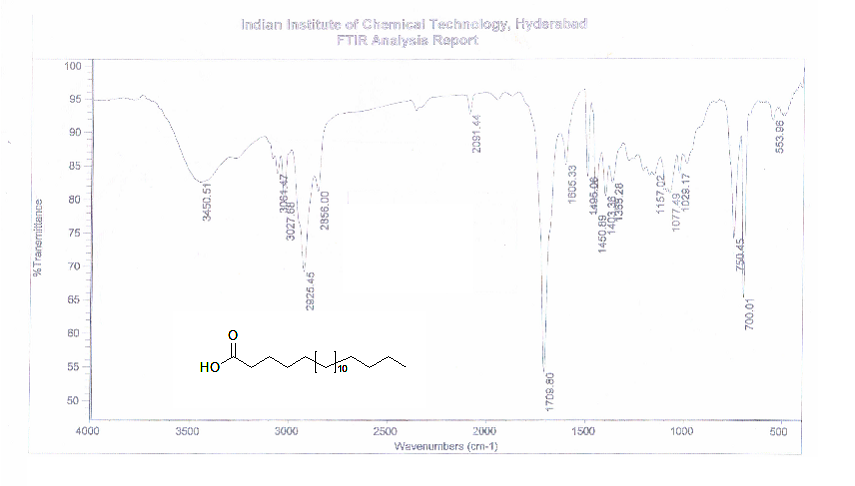


**Fig. S5a** FTIR spectrum of the compound D2Bb produced by *Rhodococcus sp.* VLD 10


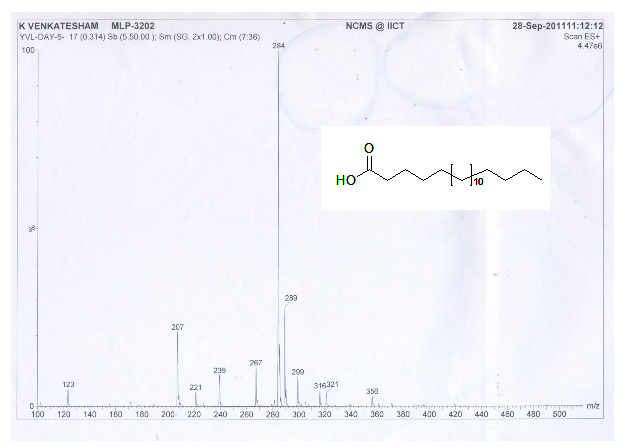


**Fig. S5b** Mass spectrum of the compound D2Bb produced by *Rhodococcus* sp. VLD 10


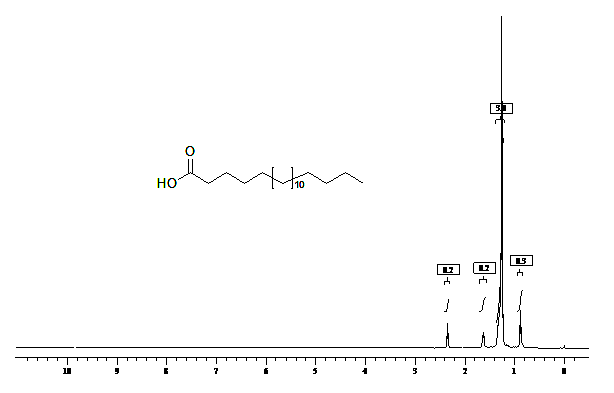


**Fig. S5c** ^1^H NMR spectrum of compound D2Bb produced by *Rhodococcus* sp. VLD 10


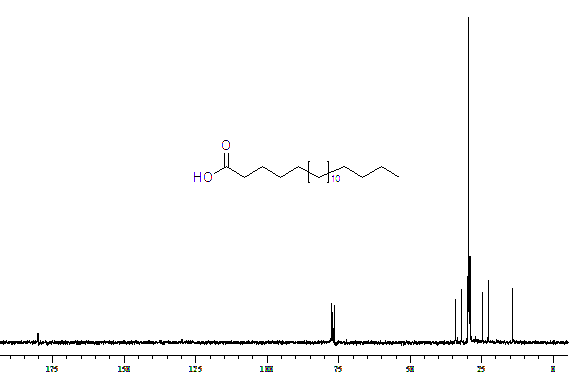


**Fig. S5d** ^13^C NMR spectrum of compound D2Bb produced by *Rhodococcus* sp. VLD 10


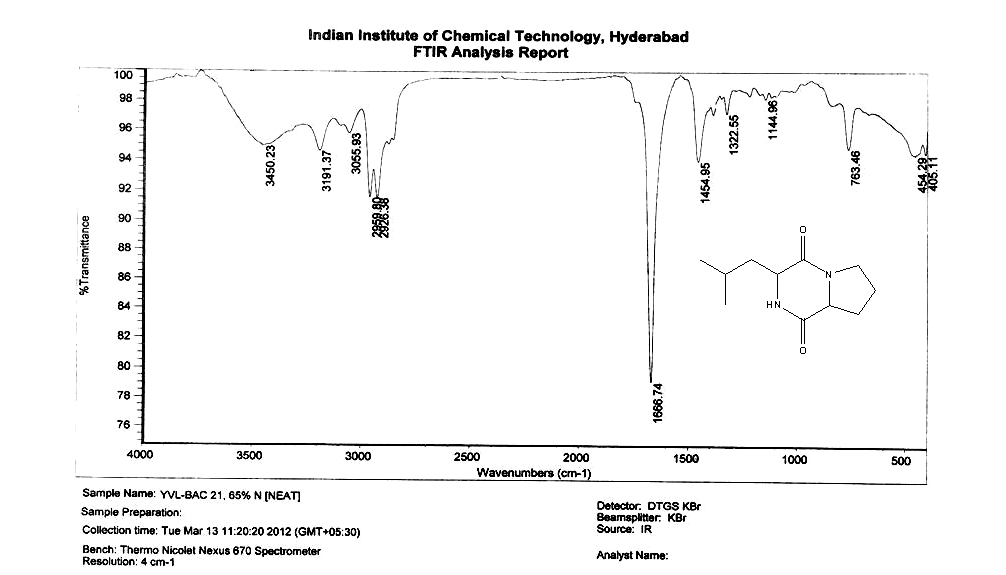


**Fig. S6a** FTIR spectrum of the compound D3 produced by *Rhodococcus* sp. VLD 1**0**


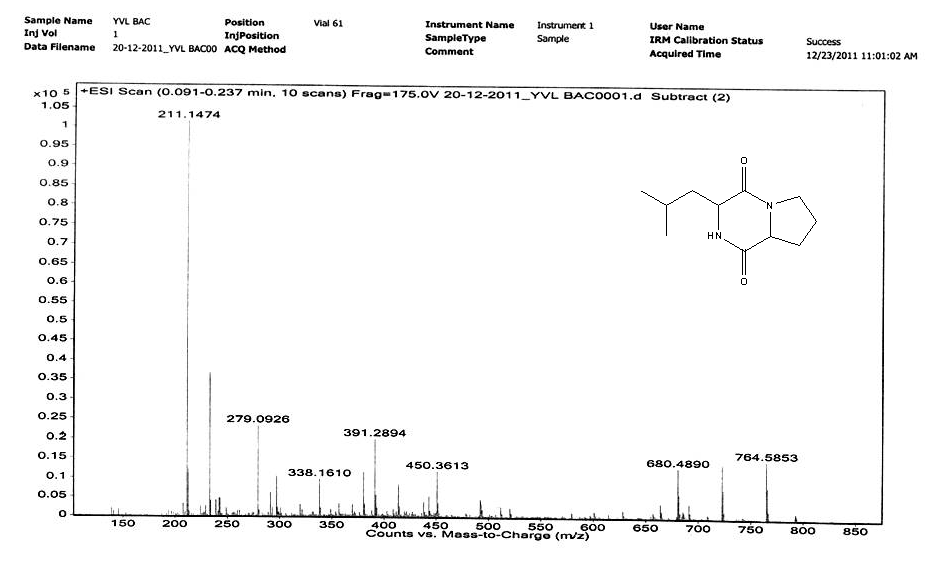


**Fig. S6b** Mass spectrum of the compound D3 produced by *Rhodococcus* sp. VLD 10

**
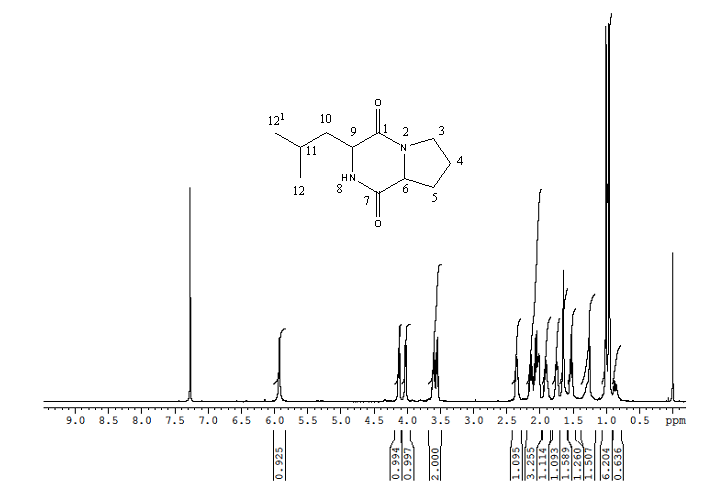
**

**Fig. S6c** ^1^H NMR spectrum of compound D3 produced by *Rhodococcus* sp. VLD 10


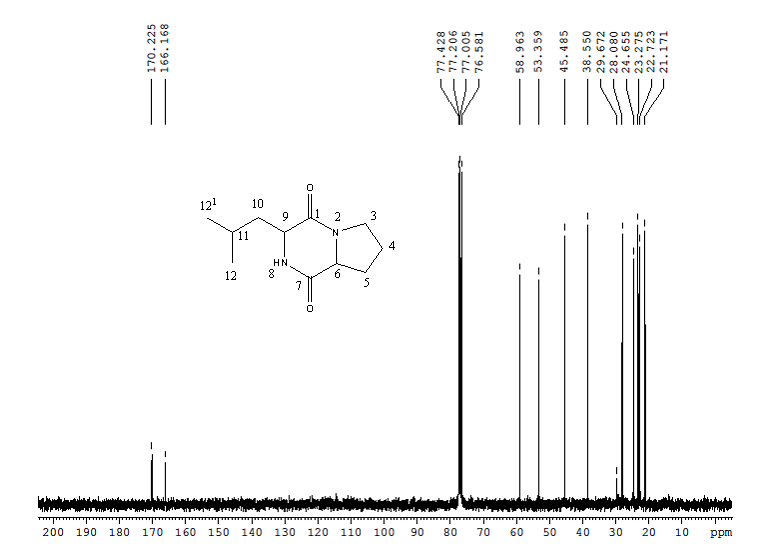


**Fig. S6d** ^13^C NMR spectrum of compound D3 produced by *Rhodococcus* sp. VLD 10


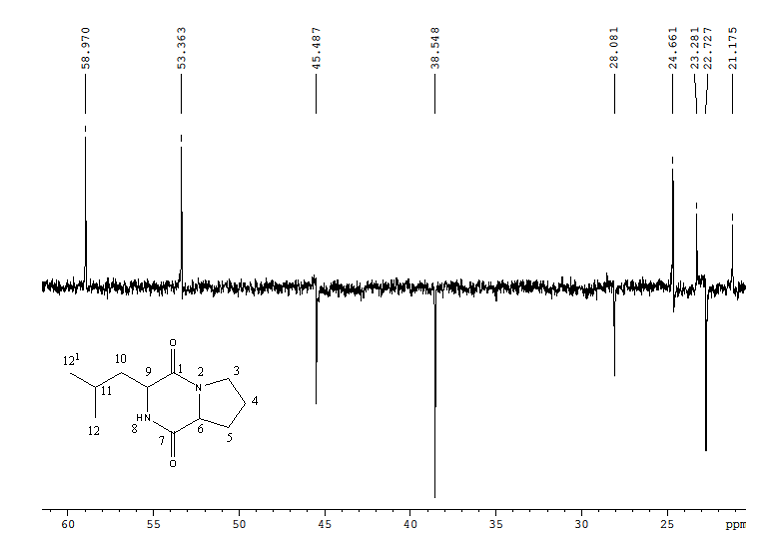


**Fig. S6e** DEPT spectrum of compound D3 produced by *Rhodococcus* sp. VLD 10


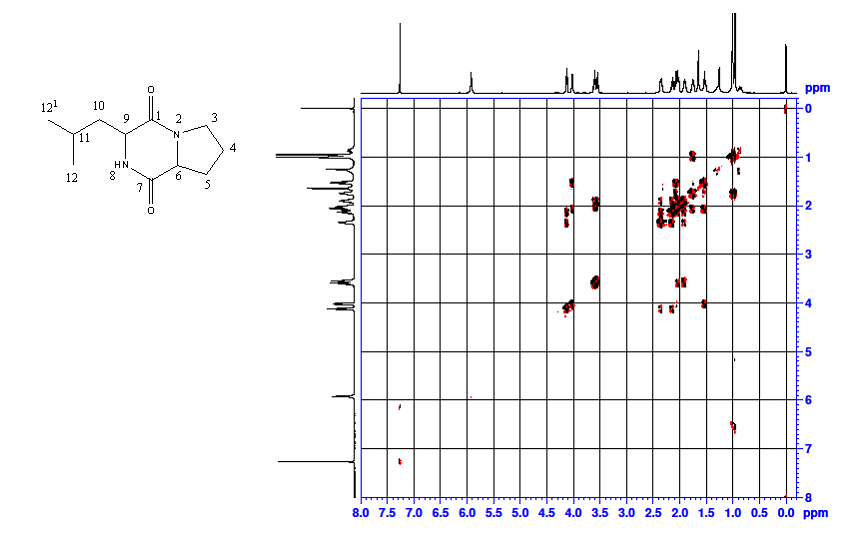


**Fig. S6f** H^1^-H^1^ COSY spectrum of compound D3 produced by *Rhodococcus* sp. VLD 10


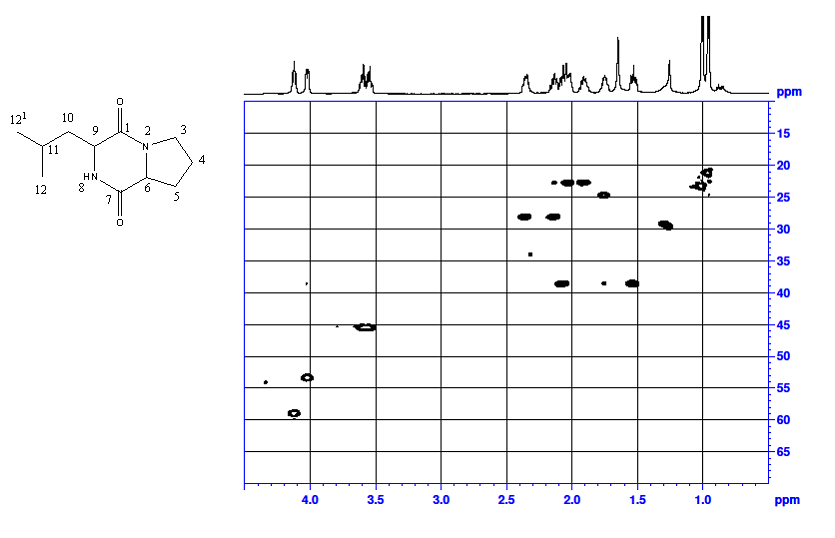


**Fig. S6g** HSQC spectrum of compound D3 produced by *Rhodococcus* sp. VLD 10


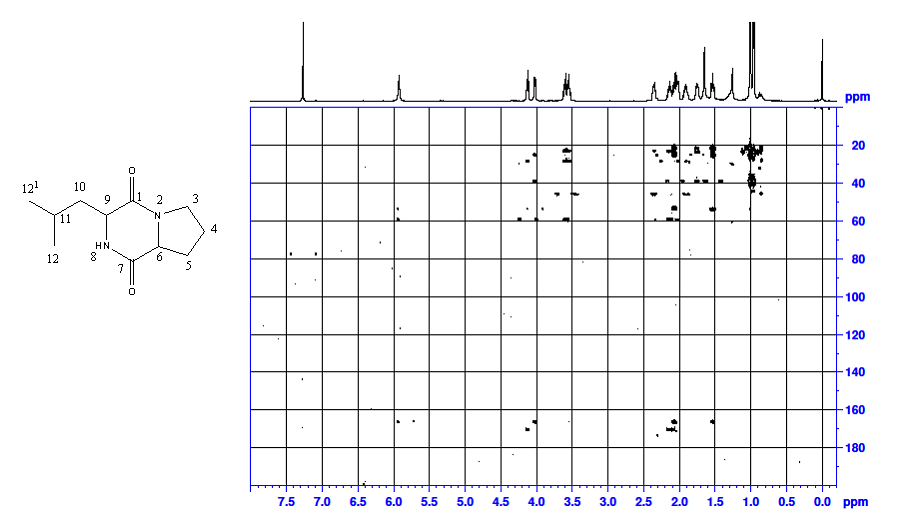


**Fig. S6h** HMBC spectrum of compound D3 produced by *Rhodococcus* sp. VLD 10
